# When perfection isn't enough: host egg signatures are an effective defence against high-fidelity African cuckoo mimicry

**DOI:** 10.1098/rspb.2023.1125

**Published:** 2023-07-26

**Authors:** Jess Lund, Tanmay Dixit, Mairenn C. Attwood, Silky Hamama, Collins Moya, Martin Stevens, Gabriel A. Jamie, Claire N. Spottiswoode

**Affiliations:** ^1^ DST-NRF Centre of Excellence at the FitzPatrick Institute of African Ornithology, University of Cape Town, Rondebosch, Cape Town, South Africa; ^2^ Department of Zoology, University of Cambridge, Cambridge, UK; ^3^ Musumanene Farm, Choma, Zambia; ^4^ Centre for Ecology and Conservation, University of Exeter, Penryn Campus, Penryn TR10 9FE, UK

**Keywords:** perfect mimicry, avian brood parasitism, egg signatures, coevolution

## Abstract

Most mimicry systems involve imperfect mimicry, whereas perfect and high-fidelity mimicry are rare. When the fidelity of mimicry is high, mimics might be expected to have the upper hand against their antagonists. However, in coevolving systems, diversification of model phenotypes may provide an evolutionary escape, because mimics cannot simultaneously match all model individuals in the population. Here we investigate high-fidelity mimicry in a highly specialized, Afrotropical brood parasite–host system: the African cuckoo and fork-tailed drongo. Specifically, we test whether host egg polymorphisms are an effective defence against such mimicry. We show, using a combination of image analysis, field experiments and simulations, that: (1) egg colour and pattern mimicry of fork-tailed drongo eggs by African cuckoos is near-perfect on average; (2) drongos show fine-tuned rejection of foreign eggs, exploiting unpredictable pattern differences between parasitic eggs and their own; and (3) the high degree of interclutch variation (polymorphic egg ‘signatures’) exhibited by drongos gives them the upper hand in the arms race, with 93.7% of cuckoo eggs predicted to be rejected, despite cuckoos mimicking the full range of drongo egg phenotypes. These results demonstrate that model diversification is a highly effective defence against mimics, even when mimicry is highly accurate.

## 1. Introduction

Evolutionary arms races between models and mimics can arise when models of deceptive mimics suffer a cost from being mimicked [1]. Such a cost arises when receivers treat models as mimics, or mimics as models [2]. In some coevolving systems, mimicry can be perfect, defined as a lack of consistent differences between model and mimic phenotypes. Yet models of perfect mimics may not have lost the arms race against their mimics, because diversifying selection on model phenotypes might make it impossible for mimics to fool all receiver individuals all of the time, even if the mimic evolves highly accurate and similarly diverse phenotypes to its model. Here we ask whether diversifying selection can provide an escape route for models of high-fidelity mimics, by studying a high-fidelity, coevolved mimicry system: egg mimicry by the brood-parasitic African cuckoo *Cuculus gularis* of its host the fork-tailed drongo *Dicrurus adsimilis*.

Diversifying selection on models to escape mimicry can lead to the evolution of polymorphic ‘signatures’ of identity among individuals of the same species of model. Signatures are individually distinctive phenotypes that vary between but are consistent within individuals, meaning that an individual mimic cannot be a good match to the entire model population [3–5]. Mimics will therefore be mismatched to at least some model phenotypes. Examples of signatures typically come from systems in which receiver and model are the same species and thus need to distinguish self (i.e. the model) from non-self (i.e. the mimic) [6]. For example, some social insects have evolved diverse hydrocarbon signatures on their cuticles that provide signals of species or colony identity [7], which may be used as a defence against parasites with mimetic hydrocarbons [5]. Similarly, in the human immune system, diversification in antigen-presenting glycoproteins (encoded by major histocompatibility complex genes) generates signatures of self against which pathogens can be discriminated [8].

Polymorphic signatures of individual identity are widespread in avian brood parasite-host systems [9,10]. In these systems the brood parasite foists the costs of raising its offspring onto another species (the host) by laying its eggs in the host's nest. In some systems, selection on hosts to recognize and reject foreign eggs has led to the evolution of parasitic mimicry [10]. In turn, some hosts have evolved signatures of identity on their eggs, incorporating colour and pattern traits [4,11–15]. Host females lay a consistent egg type throughout their life [16], and so can reject eggs with phenotypes that differ from this egg signature [17–20]. The outcome of a parasitism event is typically strongly determined by the degree of matching between the parasite ‘forgery’ and the host signature [17,21], and consequently some parasites of polymorphic hosts have evolved polymorphic eggs themselves [9,21–23]. Yet even if this occurs, host signatures reduce the chance that a parasitic forgery will closely match any one host's signature. For example, the tawny-flanked prinia lays polymorphic eggs which are mimicked by the polymorphic cuckoo finch [21], but a cuckoo finch female will likely only be successful if she happens to lay an egg in a host clutch that matches her own. This illustrates that host signatures function as a defence against parasitism in multiple systems (reviewed by [6]), and are effective against imperfect mimics [21,24]. Accordingly, egg signatures have evolved independently in many host families of avian brood parasites [11,14–18,25–27]. However, to our knowledge, no study has investigated how effective signatures may be as a defence in systems with highly accurate mimicry.

To address this gap we studied an Afrotropical system in which mimicry is highly accurate: the fork-tailed drongo *Dicrurus adsimilis* and its specialist parasite, the African cuckoo *Cuculus gularis*. Both the cuckoo and its drongo host show a high degree of variability in egg phenotype between females: egg ground colour varies from white to reddish-brown, and eggs may be immaculate or patterned with tan, brown, or black speckles or blotches. This variation is likely driven by coevolution between these two species, as is the case in other host-parasite systems [4,11–15]. Phenotypically, cuckoo and drongo eggs can broadly be grouped by colour and markings as either immaculate, speckled, blotched or erythristic, although these are not discrete polymorphisms and intermediate phenotypes occur (cluster analysis in electronic supplementary material §5, electronic supplementary material, figure S2). To the human eye, population-level variation in African cuckoo egg phenotypes overlaps completely with that of fork-tailed drongo eggs, and some cuckoo eggs are indistinguishable to us until hatching.

Here, we test whether diversified egg signatures in drongos are an effective defence against such high-fidelity mimicry by cuckoos. To do so we quantify three key components of this system: (1) the mimetic fidelity of the system, (2) egg rejection behaviour by fork-tailed drongo hosts and (3) the probability of a cuckoo egg avoiding rejection in the population. (1) *Mimetic fidelity*: We measure mimetic fidelity by comparing colour (using spectrophotometry) and pattern (using image analysis) between parasite and host eggs. If mimicry is perfect at the population level then there should be no mean differences in colour or pattern between the two species, and discrimination models should be unable to differentiate between species due to a complete overlap in phenotypic space. (2) *Rejection by drongos*: We investigate what decision rules hosts use to detect parasitic eggs by conducting egg discrimination experiments in the field. (3) *Probability of cuckoo egg acceptance*: We combine our quantification of African cuckoo and fork-tailed drongo egg phenotypes with our experimental data to simulate drongo egg rejection behaviour of eggs in the cuckoo population. This analysis yielded a predicted probability that a given cuckoo egg would be rejected from a randomly selected drongo nest. Together, our analyses show that a combination of diversified egg signatures and egg rejection by hosts is a highly effective defence against what we find to be near-perfect mimicry by the African cuckoo.

## 2. Material and methods

### (a) Study site and system

**Figure 1.**
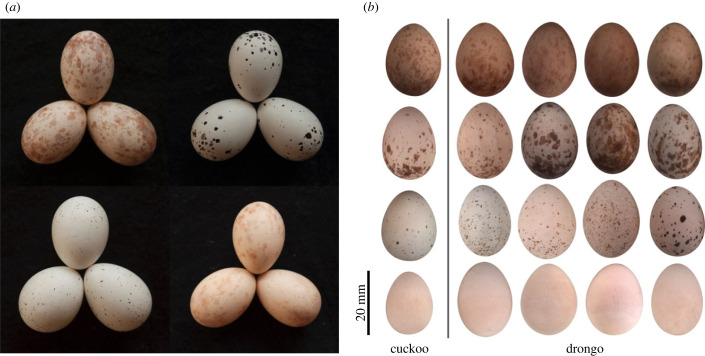
Fork-tailed drongo and African cuckoo eggs from the study site. (*a*) Naturally parasitised drongo clutches from the historical egg collection of Major John Colebrook-Robjent, collected at the study site and deposited at Livingstone Museum, Zambia. In each clutch, the cuckoo egg is on the bottom right. (*b*) Cuckoo eggs (left) and drongo eggs (right) photographed during the study period. Each egg belongs to a different female. Each egg was randomly selected from each of the four broad phenotypic categories (from top to bottom): erythristic, blotched, speckled, immaculate (assigned to group with a combination of manual sorting and cluster analysis, electronic supplementary material, §5).

Field work took place in September–November 2009, 2010, 2011 and 2019 within an approximately 3500 hectare area centred at 16°45′S, 26°54′E in the Choma district, southern Zambia. The habitat is a mixture of miombo (deciduous) woodland and agricultural fields including tobacco and maize cultivations, and pasture. Drongo nests were located and accessed by a team of field assistants living on the farms making up the study site. Fork-tailed drongos are abundant, and the parasitism rate by African cuckoos was 17.3% in 196 drongo nests across the 4 years during which we conducted experiments (25.9%, 10.3%, 23.8% and 14.3% in 2009, 2010, 2011 and 2019, respectively). This is likely an underestimate as cuckoo eggs that are poor matches to host clutches may have been rejected before we found the nests. To the human eye, African cuckoo eggs look very similar in colour and markings to drongo eggs, as well as being similar in size (figure 1). Female drongos lay a single egg type throughout their life, and so our egg phenotypic metrics were highly consistent within clutches (intraclass correlation coefficient for all colour and pattern measures > 0.69, *p* < 0.001; electronic supplementary material, table S1). They readily reject dissimilar eggs from their nest [28]. We detected parasitism events by the presence of an egg differing from other eggs in the clutch in pattern or colour, or shape (cuckoo eggs often have a blunter wide pole). Cuckoo eggs could be challenging to detect to the human eye, and we occasionally overlooked a cuckoo egg until it hatched.

Clutches laid by the same drongo female, and eggs laid by the same cuckoo female, were identified based on location and similarity in phenotypes. Only one egg per drongo or cuckoo female was analysed, unless otherwise stated.

### (b) Are African cuckoo eggs perfect mimics of fork-tailed drongo eggs?

Mimetic fidelity based on traits of models and mimics tends to be measured by calculating some measure of overlap in (or difference between mean values of) model and mimic traits, across the model and mimic populations [21,24,29–31]. For the cuckoo and drongo system, we measured mimetic fidelity by quantifying drongo and cuckoo egg phenotypes using established methods that yield biologically relevant measures of colour and pattern (see below).

#### (i) Eggshell colour mimicry

Eggshell background colour (i.e. avoiding pattern markings) was measured using spectrophotometry following [21] (see electronic supplementary material, §2 for details). To account for the visual system of drongos, reflectance spectra were converted to ‘cone catch’ values in Pavo [32,33] by calculating the predicted cone catches over wavelengths 300–700 nm of each of the four single cone types (UV-sensitive, shortwave-sensitive, mediumwave-sensitive and longwave-sensitive, encoding colour information [34]), and double cones (thought to encode luminance information [35]). The spectral sensitivities of the Indian peafowl *Pavo cristatus* were used to model stimulation of the cones [36] because, like peafowl, drongos have violet-sensitive vision [37]. Standard daylight (D65) illuminance was used to calculate irradiance because drongo nests are well-lit (shallow open cups, often in direct sunlight).

In addition to comparing the cone catches for each cone type between cuckoos and drongos, we also calculated the centroid of the cuckoo population and the centroid of the drongo population in tetrahedral colour space. To test whether these colours would be discriminable, we calculated the chromatic and achromatic contrasts (measured in just noticeable differences, JNDs) between the two. JND values below one indicate that two colours are not discriminable [38,39].

#### (ii) Eggshell pattern mimicry

Eggshell pattern was quantified from photographs of eggs taken under natural light in RAW format. Images were normalized for light conditions, and standardized to 19 px/mm in ImageJ using the DCRAW plugin [40], following [21] with minor alterations (see electronic supplementary material, §2 for details). We quantified eggshell pattern from these images using three methods: (i) adaptive thresholding (implemented in ImageJ [41]), (ii) the granularity approach (implemented using the MICA Toolbox in ImageJ, following [41,42]) and (iii) NaturePatternMatch (NPM) [14,43]. Adaptive thresholding classifies regions of the egg as ‘pattern’ or ‘background’ to measure the distribution of pattern over an egg, the granularity approach measures the spatial scale of light and dark components of pattern, and NPM measures the shape and orientation of individual markings using SIFT (scale-invariant feature transform) analysis [44]. These methods yielded five measures of pattern: pattern coverage (a combined measure of how much of the egg is covered in markings and the distribution of those markings), PC energy (the first principal component of pattern contrast and variability in contrast), number of features and mean feature size (see electronic supplementary material, §2 for detailed methods and explanations of pattern measures).

#### (iii) Egg size and shape

Egg length and width were measured with digital calipers. Egg shape was calculated as the width : length ratio.

#### (iv) Statistical analysis

We tested whether egg traits (colour, pattern, size and shape) differed between cuckoo and drongo eggs using Wilcoxon rank-sum tests. Because immaculate eggs yield unreliable measures of mean feature size, we restricted our analysis of drongo and cuckoo pattern to patterned eggs (*n* = 166 and 26, respectively). Three drongo eggs had extremely high measures of mean feature size that were not representative of the actual marking size of the egg, suggesting they were artefacts of shadows. They were identified as outliers according to Mahalanobis distance and excluded from tests for differences (conclusions were unaffected).

#### (v) Discriminant function analysis

To assess the overall degree of phenotypic matching between cuckoos and drongos, we used discriminant function analysis (DFA [45]) to assess the likelihood of an observation being assigned to the correct group (cuckoo or drongo) based on egg colour and pattern traits. All DFAs had equal (i.e. uninformative) priors and were implemented using the *lda* function in R [46,47]. We used DFA rather than multinomial logistic regression (MLR) because DFA is more powerful when its assumptions of normality and equal variance are met [48], which they were here (electronic supplementary material §4a). We used jack-knifed (leave-one-out) predictions to remove the influence of differences in sample size between species [49]. Outliers were identified according to Mahalanobis distances [50] and removed, although retaining outliers did not significantly influence the accuracy of the DFAs. For the DFA we chose a subset of egg traits (pattern dispersion, total energy, feature number, MW cone catch and luminance) which were not strongly collinear (*r* < 0.7; electronic supplementary material, table S8).

As an additional check, we also performed a second version of the DFA by taking the first three principal components of a principal component analysis (PCA) based on ten egg traits (see electronic supplementary material, §4a).

To situate the cuckoo–drongo system relative to another brood parasite–host system, we also tested the performance of a discriminant function analysis on a system with known imperfect mimicry: the tawny-flanked prinia *Prinia subflava* and its cuckoo finch *Anomalospiza imberbis* host-race [21] (see electronic supplementary material, §4b). For each version of the DFA, we used Fisher's exact tests to assess whether DFA performed better than chance at assigning egg observations to the correct species.

### (c) How do drongos detect foreign eggs?

To investigate egg rejection by drongos as a defence against cuckoo parasitism, we performed egg rejection experiments in the field. This enabled us to build a model of drongo rejection based on phenotypic traits of eggs, which we could then use to predict their responses to real cuckoo eggs (discussed in next section).

#### (i) Egg rejection experiments

Egg rejection experiments on fork-tailed drongos were performed in 2009, 2010, 2011 and 2019 (*n* = 14, 19, 19 and 62 respectively). To simulate a parasitism event, we replaced a host egg with a conspecific egg from another female (hereafter ‘experimental egg’; a surrogate for a cuckoo egg), which mimics the laying behaviour of a female cuckoo ([51] and pers. obs. from our field site). The high degree of similarity between cuckoo and drongo eggs makes using conspecific eggs a reasonable protocol, which we validated with *post-hoc* simulations (electronic supplementary material, §1). Handling of eggs likely does not influence rejection behaviour (electronic supplementary material, §3).

Because drongos are highly discerning hosts, where possible we provided them with difficult rejection decisions by adding an experimental egg with a similar phenotype to the host clutch, although the overall dataset included a similar range of host–parasite mismatches as drongos encounter from real cuckoo eggs. We gave drongos difficult decisions where possible because randomly selecting experimental eggs to place in host nests would have resulted in very few acceptances, due to high drongo rejection sensitivity (only 10 of 114 experimental eggs would have been accepted based on simulation results, see §3c). This would not have provided enough power to build a model of egg rejection, since even with our protocol of giving drongos difficult decisions, only a third of experiments resulted in the experimental egg being accepted (see electronic supplementary material, §3b). Non-random assignment of experimental eggs to host nests should not bias the model towards higher or lower thresholds for egg rejection, as the threshold for rejection remains the same regardless of the distribution of differences in parasite and host eggs.

We monitored experiments for four days, daily in 2019 and daily when possible in 2009–2011, to determine whether the experimental egg was rejected or accepted. We took the disappearance of the experimental egg to be evidence of host rejection. In six experiments, an egg from the host clutch was rejected either alone (*n* = 4) or as well as the experimental egg (*n* = 2). If the nest was depredated or destroyed within the first four days (identified by all eggs missing from the nest or the nest being broken), the experiment was excluded.

#### (ii) Modelling rejection by drongos

Pattern traits of each host and experimental egg were quantified following the methods in §2b. Background colour is highly repeatable within a drongo clutch (electronic supplementary material, table S1), so colour of the removed egg was taken as representative of the clutch. Drongo rejection of experimental eggs was modelled with generalized linear models (binomial response variable; 0 = accepted, 1 = rejected). We ran two main models, the first (model 1) to test whether any individual traits were consistently used by drongos, and the second (model 2) which allowed a composite measure of trait distance, described below, to inform rejection decisions. The best model was selected based on AIC and was used for subsequent simulations of cuckoo parasitism (see §2d). For model 1 we used individual pattern trait differences (pattern coverage, PC energy, and number of features) as predictors. Because there appeared to be complete overlap between cuckoo and drongo egg phenotypes (i.e. no particular traits are more likely to be reliable cues of cuckoo parasitism; see §3a), we had no prior prediction for the relative importance of different phenotype attributes in egg rejection. For model 2, we calculated a multidimensional measure of pattern trait difference (multidimensional pattern distance): the Euclidean distance between host and experimental eggs in a five-dimensional phenotypic space defined by the five pattern variables that give reliable measures for both immaculate and patterned eggs (proportion pattern, pattern dispersion, total energy, standard deviation energy and number of features, but excluding mean feature size, which yields erroneous values for immaculate eggs).

A separate model including all two-way interactions showed no evidence for any interaction terms (electronic supplementary material, table S7). Including colour (chromatic colour contrasts between host and experimental eggs; measured in JNDs and calculated in PAVO [33]) as a cue for rejection by drongos in model 1 and model 2 did not improve the AIC of either model (electronic supplementary material, tables S5 and S6) and was therefore excluded from subsequent analysis.

We checked for multicollinearity in models with multiple predictors using the *vif* function in the car package [52]. Adjusted *R*^2^ (using the *rsq* function in R) was used to determine the proportion of variance explained by models, and we partitioned variance explained (%I) between predictors [53] using the hier.part package [54]. We used likelihood ratio tests to compare nested models using the *lrtest* function in the epiDisplay R package [55].

### (d) How often do we expect a cuckoo's egg to be accepted in nature?

To estimate how often a laying female cuckoo would have her egg accepted, we simulated parasitism events using phenotypes of drongo and cuckoo eggs. Over 1000 iterations, we selected (with replacement) a drongo egg and a cuckoo egg and calculated the multidimensional pattern distance between them. We then used the best-supported generalized linear model of rejection of conspecific eggs (model 2; rejection∼difference in multidimensional pattern distance) to predict whether this simulated parasitism event would be successful (cuckoo egg accepted) or unsuccessful (cuckoo egg rejected).

### (e) How often do we expect a cuckoo's egg to be accepted in hypothetically monomorphic cuckoo and drongo populations?

The simulations detailed in §2d above revealed a very high level of rejection (93.7%; see §3c). To determine whether this high rejection rate was due specifically to signatures, rather than any average host-parasite differences, we estimated the probability of rejection in a hypothetical population of monomorphic drongos and cuckoos. The phenotypes of the hypothetical monomorphic drongo and cuckoo populations were calculated as the centroids in multidimensional phenotypic pattern space for each species. We substituted this value into the model of rejection (model 2) to calculate the likelihood that a cuckoo egg in a hypothetical monomorphic population would escape rejection by a drongo in hypothetical monomorphic population. If such a cuckoo egg would generally be accepted by a hypothetical monomorphic drongo, this would provide evidence that it is signatures, rather than some other property of drongo and cuckoo egg phenotypes, that drive the high rejection rate (93.7%; see §3c) found in the parasitism simulations above. These drongo and cuckoo hypothetical egg morphs do not necessarily exist in nature and the goal of this analysis is to compare these hypothetical rejection rates to predicted rejection rates for real cuckoo and drongo populations (which are both polymorphic).

We conducted all statistical analyses in R v. 4.2.1 [56].

## 3. Results

### (a) Are African cuckoo eggs perfect mimics of fork-tailed drongo eggs?

**Figure 2.**
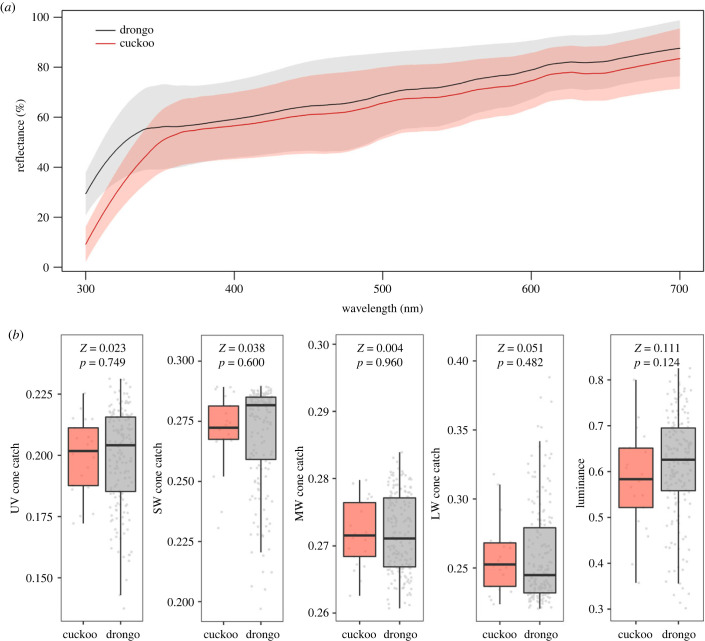
Colour mimicry. (*a*) Objective colour of cuckoo and drongo eggs: mean spectral reflectance curves of the background colour of African cuckoo eggs (red) and fork-tailed drongo eggs (grey). Shading indicates 95% confidence intervals. (*b*) Avian-perceived colour of cuckoo and drongo eggs: relative cone catch differences of each of the single cone types (ultraviolet-sensitive, UV; shortwave-sensitive, SW; mediumwave-sensitive, MW; and longwave-sensitive, LW) and the double cones (luminance) for the ground colour of cuckoo eggs (*n* = 21) and drongo eggs (*n* = 171) found in 2009–2011 and 2019. Statistical values are from Wilcoxon rank-sum tests. Whiskers indicate 1.5 × interquartile ranges.

We objectively measured egg background colour of drongo and cuckoo eggs using mean spectral curves of drongo and cuckoo eggs. These matched each other closely at wavelengths from 350–700 nm, but drongos had higher reflectance at shorter UV wavelengths from approximately 300–315 nm (figure 2*a*). Egg colour as a drongo would perceive it (measured as the relative cone catches of the four single cone types and double cones) did not differ significantly between cuckoo and drongo eggs (figure 2*b*). Furthermore, the chromatic and achromatic contrasts between the centroid of cuckoo colour space and the centroid of drongo colour space were 0.12 JNDs and 0.56 JNDs respectively. Since JND values below 1 indicate that two colours should not be discriminable by drongos [38,39], the colour of an ‘average’ cuckoo egg in the population is not discriminable from the colour of an ‘average’ drongo egg. Taken together these results suggest that from a drongo's visual perspective, colour mimicry by cuckoos is effectively perfect.

**Figure 3.**
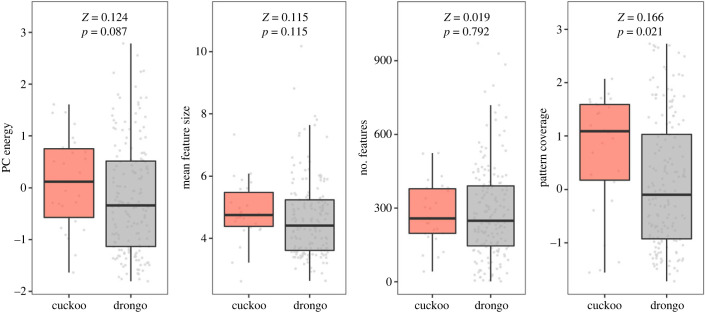
Pattern mimicry. Pattern differences between cuckoo (*n* = 26) and drongo (*n* = 166) patterned eggs (excluding immaculate eggs, which yield erroneous measurements of mean feature size). Cuckoos had significantly higher pattern coverage (a synthetic measure of pattern proportion and dispersion), but did not differ in PC energy (a synthetic measure of pattern contrast and variability), mean feature size, or number of features. Statistical values are from Wilcoxon rank-sum tests. For mean feature size, three drongo eggs were excluded as outliers, but this did not influence the conclusion. Whiskers indicate 1.5 × interquartile ranges.

Drongo and cuckoo egg pattern did not differ significantly in PC energy, mean feature size or number of features. However, pattern coverage (a synthetic measure incorporating both how concentrated pattern is at a pole of an egg and how much of the egg is covered by markings) was significantly higher in cuckoos than in drongos (figure 3), probably because blotched eggs, which had the highest pattern coverage, were more frequent in the cuckoo population than the drongo population (see electronic supplementary material, §5). This indicates that overall, pattern mimicry by African cuckoos is near-perfect.

In the drongo and cuckoo system, discriminant function analysis (DFA) based on raw colour and pattern measurements (pattern dispersion, total energy, feature number, MW cone catch and luminance) showed that cuckoo eggs were correctly assigned to species 65.0% of the time, and drongo eggs were correctly assigned 67.1% of the time. This DFA performed significantly better than chance at assigning observations to species, but the effect size was small (Fisher's exact test, odds ratio = 3.75, *p* = 0.007). When the DFA was repeated with principal components as informants, the discriminant function did not perform better than chance (electronic supplementary material §4a). By contrast, in the cuckoo finch and tawny-flanked prinia system (a known case of imperfect mimicry) flexible discriminant analysis performed well, and correctly assigned cuckoo finches to species 87.2% of the time, and prinias, 72.7% of the time (electronic supplementary material, §4b). Together, the results of these DFAs show that mimetic fidelity of the cuckoo-drongo system is high.

In summary, at the population level, egg phenotypes of fork-tailed drongos and African cuckoos matched across nearly all traits measured, differing significantly only in pattern coverage. Discriminant function analyses show that mimicry is highly accurate: DFAs performed marginally better than chance at assigning observations to species, driven by the slightly higher pattern coverage in cuckoo eggs than drongo eggs on average (figure 3; omitting this trait meant the model performed marginally worse than chance, *p* = 0.053). This suggests that while pattern mimicry is not perfect, it is nonetheless highly accurate.

Egg length, width and shape did not differ between drongos and cuckoos (electronic supplementary material, table S9).

### (b) How do drongos detect foreign eggs?

We conducted 114 egg rejection experiments on fork-tailed drongos, of which 38 resulted in acceptance and 76 in rejection of the experimental egg.

To test whether any one pattern trait consistently explained egg rejection, we ran a model with pattern coverage, PC energy, and number of features as predictors of egg rejection (model 1; see §2c for why these traits were used). Only pattern coverage and the number of features were significant predictors, and most of the variance was explained by differences in feature number (%I = 52.8; electronic supplementary material, table S3). This model (model 1) explained slightly less variation (adjusted *R*^2^ = 27.2%) than a model with only multidimensional pattern distance as a predictor (model 2; adjusted *R*^2^ = 29.6%), and had a higher AIC (ΔAIC = 5.4; electronic supplementary material, table S4). We therefore used model 2 for subsequent simulations predicting how often a cuckoo egg would be accepted in nature (§3c).

### (c) How often do we expect a cuckoo's egg to be accepted in nature, and in hypothetically monomorphic cuckoo and drongo populations?

We simulated 1000 parasitism events by real cuckoo eggs in the local population, and predicted rejection based on the best model (model 2; rejection∼multidimensional pattern distance). The predicted rate of rejection was extremely high (93.7%).

To confirm that the results above were driven by the existence of signatures (rather than any average differences between cuckoo and drongo eggs), we created the hypothetical phenotypes of cuckoo and drongo populations if both species were monomorphic. These phenotypes were calculated as the centroid in multidimensional pattern space for each species independently. The multidimensional pattern distance between a cuckoo egg in a hypothetically monomorphic population and a drongo egg in a hypothetically monomorphic population was 0.75 (dimensionless). Based on the model used for the simulations (model 2), the probability of rejection in this hypothetical scenario is 35.9%. Thus, if both populations were monomorphic, a cuckoo egg would generally be accepted in a drongo clutch.

## 4. Discussion

In this study, we show that although African cuckoo eggs are near-perfect mimics of fork-tailed drongo eggs, diversification of drongo egg ‘signatures’ gives drongos the upper hand in their co-evolutionary arms race. This effect arises because of frequent mismatches between a given cuckoo egg phenotype and the highly diverse individual egg phenotypes (signatures) of drongo eggs, demonstrating that such signatures are a highly effective defence against a high-fidelity mimic. Drongos are thus able to correctly reject a far higher proportion of cuckoo eggs (estimated at 93.7% of cuckoo laying events) than would be otherwise expected given the extreme similarity between ‘average’ cuckoo and drongo egg phenotypes.

### (a) Do African cuckoos perfectly mimic fork-tailed drongo eggs?

The ground colours of cuckoo and drongo eggs were extremely similar, and modelled stimulation of colour and double cones showed that, from the drongo's perspective, cuckoo and drongo eggs do not differ at the population level. Similarly, egg pattern differed very little between cuckoos and drongos at the population level. Only pattern coverage (a synthetic metric incorporating information about the proportion of the eggshell covered by markings, and the distribution of those markings) differed significantly between cuckoos and drongos. This difference probably arose from the higher proportion of blotched eggs in the cuckoo population compared to the drongo population (electronic supplementary material §5, electronic supplementary material, figure S3). The similarity in cuckoo and drongo egg pattern contrasts with many other brood parasite–host systems, where there are significant differences in multiple pattern attributes, including those analogous to the pattern measures quantified here, and detectable even in smaller samples [21,57–59].

Comparing mimetic fidelity in the cuckoo-drongo system to that of another system further highlights the accuracy of African cuckoo mimicry. The tawny-flanked prinia and cuckoo finch system is a known case of imperfect mimicry [21,24] and flexible discriminant analysis correctly assigned cuckoo finches to species 87.2% of the time, and prinias, 72.7% of the time (electronic supplementary material, §4b). This contrasts with the cuckoo and drongo system where discriminant function analysis only correctly assigned cuckoos and drongos to species 65.0% and 67.1% of the time respectively. This confirms that the mimetic fidelity of the cuckoo finch–prinia system is lower than that of the cuckoo-drongo system.

Taken together, these results show that drongos are faced with a mimic that, at the population level, is nearly indistinguishable from themselves. This implies that a consistent rejection rule involving specific traits (cf. [21,43,57,59]) would perform poorly in this system because no single trait is consistently informative at the population level, since no single trait provides a strongly reliable cue of parasitism across all potential host-parasite combinations.

### (b) How do drongos detect foreign eggs?

In accordance with the expectation that rejection rules consistently involving specific traits would perform poorly in this system, we found little evidence that drongos consistently used any single trait as a cue to reject foreign eggs. Instead, the best predictor of egg rejection was a multidimensional measure of pattern distance, which synthesizes differences in multiple pattern traits. The key difference between multidimensional pattern distance and individual trait differences is that an elevated multidimensional pattern distance between eggs can result from large differences in any one or more of its component traits. Thus, our finding that multidimensional pattern distance is the best predictor of rejection suggests that different traits are informative in different individual parasitism events, depending on the individual host and parasitic phenotypes involved. This implies that drongos were able to evaluate several potential cues, and act on any differences arising from this set of potentially informative (but on average uninformative) traits. This contrasts with other systems in which specific traits consistently provide reliable cues of parasitism, and appear to be used as such [21,43,57,59].

### (c) How often do we expect a cuckoo's egg to be accepted in nature, and in hypothetically monomorphic cuckoo and drongo populations?

Our results suggest that despite near-perfect mimicry of drongo eggs at the population level, African cuckoos do not have the upper hand in the arms race. To quantify this, we substituted phenotypic values of cuckoo eggs from our population into our egg rejection model, which simulates the fate of potential parasitism events. These simulations predicted that drongos reject real cuckoo eggs from their nests in 93.7% of parasitism events; thus, despite near-perfect mimicry, cuckoo eggs have a high probability of being rejected.

Given that mimicry is near-perfect, we hypothesized that rather than any average host-parasite difference driving high rejection rates, it is inter-individual variation (i.e. signatures) which explains this pattern. To confirm this, we conducted a simulation to predict the rejection rate in a hypothetically monomorphic drongo population. As predicted, we found that the probability of a cuckoo egg being rejected in this hypothetical population is lower than in the real populations (35.9% and 93.7% respectively), illustrating the important role of signatures in driving the high observed and predicted rejection rate in this system. Specifically, signatures in the drongo population result in a low likelihood of a randomly laid cuckoo egg being a sufficiently good match to avoid rejection by drongos. This highlights the importance of considering variability in phenotypes, and not only their population averages, in understanding their adaptive role.

The results of the simulated parasitism events only approximate natural rejection probabilities, given that sample size of independent cuckoo eggs was relatively small (*n* = 28). Thus, the precise values of estimates should be interpreted with caution. However, the comparison between simulated rejection rates for the natural populations and hypothetical monomorphic populations is likely robust, given that the same cuckoo population is considered in both simulations.

### (d) How do African cuckoos cope with high rejection rates?

The diverse egg signatures and high rates of rejection in this system contrast with hosts of the closely related common cuckoo, which show relatively low interclutch variation and weak or absent rejection [14,60,61]. This might be explained by the African cuckoo-drongo arms race being at a more advanced stage than that between the common cuckoo and its hosts. African cuckoos and drongos may have a long coevolutionary history, due to the Afrotropics having a more stable geographical history without widespread glaciation events [62,63], thereby allowing specialist interactions to persist for long periods of evolutionary time [64]. Alternatively (although not mutually exclusively), this arms race may be at an advanced stage because the African cuckoo is a specialist parasite, and specialist host-parasite interactions often show increased rates of coevolution because selection is not diffuse [65–67].

High rates of egg rejection by hosts may affect cuckoo population dynamics. If an African cuckoo lays 20 eggs per year [68,69], a female will have, on average, 1.26 eggs accepted by drongo hosts in a season (based on the predicted rejection rate of 93.7%). Given a drongo nest survival rate at our study site of 20.2% [70], this yields an expected value of 0.25 fledglings per African cuckoo female per season. This is probably an overestimate, because the simulations ignore additional cues of parasitism (such as the sight of a laying cuckoo), which probably elevate host rejection rate. This contrasts with the common cuckoo, which has a predicted success rate from laying to fledging of 27% [69], such that if a common cuckoo lays 20 eggs a year, she should expect to fledge 5.4 chicks per season. This suggests that the annual fecundity of African cuckoos is an order of magnitude lower than that of common cuckoos.

The low reproductive success in African cuckoos may contribute to a ‘slow’ life-history strategy. The lifespan of African cuckoos is unknown, but common cuckoos (which are similar in size, but have a potentially more costly trans-Saharan rather than intra-African migration [71]) have a lifespan of up to seven years [72]. Tropical species tend to live longer than their temperate relatives [73]; if we therefore conservatively estimate that an African cuckoo typically lives eight to nine years, a female could expect to fledge only two offspring during her lifetime.

At present, the African cuckoo remains a relatively common bird in sub-Saharan Africa [74], suggesting that despite the high rates of egg rejection and predation that it experiences (at least at our study site), individuals are able to produce enough successful recruits over their lifetimes that the cuckoo population remains numerous. This may be because a slow life-history strategy has evolved in tolerance of host defences [75]. Alternatively, our study site might be a coevolutionary ‘hotspot’ in a geographical mosaic of antagonistic coevolution [76], where drongo defences against parasitism are especially effective. If so, this population may act as a sink for African cuckoos, maintained by immigration from populations that interact with less well-defended drongos. Studies of population structure and of drongo-cuckoo interactions in other populations could help build a picture of the coevolutionary dynamics of this system across larger spatial scales.

## 5. Conclusion

African cuckoo eggs mimic fork-tailed drongo eggs nearly perfectly at the population level. Despite this highly accurate mimicry, fork-tailed drongos can likely recognize and reject the vast majority of cuckoo eggs, due to the high degree of variation between the eggs laid by different females (polymorphic egg signatures). This leads to a low predicted annual fecundity for individual African cuckoos (approximately one fledgling per four years per female, after taking into account nest predation), suggestive of a ‘slow’ life-history strategy. Taken together, our results demonstrate that signatures are an effective defence against a deceptive mimic, even when mimetic fidelity is high. This shows that in coevolved mimicry systems, diversification in model phenotypes may provide an evolutionary escape for models, counterbalancing even high-fidelity mimicry.

## Data Availability

Data and R code have been uploaded to Dryad [77]. Supplementary methods and results are provided in electronic supplementary material [78].
